# Genetic diversity and striatal gene networks: focus on the heterogeneous stock-collaborative cross (HS-CC) mouse

**DOI:** 10.1186/1471-2164-11-585

**Published:** 2010-10-19

**Authors:** Ovidiu D Iancu, Priscila Darakjian, Nicole AR Walter, Barry Malmanger, Denesa Oberbeck, John Belknap, Shannon McWeeney, Robert Hitzemann

**Affiliations:** 1 Department of Behavioral Neuroscience, Oregon Health & Science University, Portland OR, USA; 2 Research Service, Veterans Affairs Medical Center, Portland, OR 97239, USA; 3 Division of Biostatistics, Public Health & Preventative Medicine, Oregon Health & Science University, Portland, OR 97239, USA; 4 Division of Bioinformatics and Computational Biology, Medical Informatics & Clinical Epidemiology, Oregon Health & Science University, Portland, OR 97239, USA

## Abstract

**Background:**

The current study focused on the extent genetic diversity within a species (*Mus musculus*) affects gene co-expression network structure. To examine this issue, we have created a new mouse resource, a heterogeneous stock (HS) formed from the same eight inbred strains that have been used to create the collaborative cross (CC). The eight inbred strains capture > 90% of the genetic diversity available within the species. For contrast with the HS-CC, a C57BL/6J (B6) × DBA/2J (D2) F_2 _intercross and the HS4, derived from crossing the B6, D2, BALB/cJ and LP/J strains, were used. Brain (striatum) gene expression data were obtained using the Illumina Mouse WG 6.1 array, and the data sets were interrogated using a weighted gene co-expression network analysis (WGCNA).

**Results:**

Genes reliably detected as expressed were similar in all three data sets as was the variability of expression. As measured by the WGCNA, the modular structure of the transcriptome networks was also preserved both on the basis of module assignment and from the perspective of the topological overlap maps. Details of the HS-CC gene modules are provided; essentially identical results were obtained for the HS4 and F_2 _modules. Gene ontology annotation of the modules revealed a significant overrepresentation in some modules for neuronal processes, e.g., central nervous system development. Integration with known protein-protein interactions data indicated significant enrichment among co-expressed genes. We also noted significant overlap with markers of central nervous system cell types (neurons, oligodendrocytes and astrocytes). Using the Allen Brain Atlas, we found evidence of spatial co-localization within the striatum for several modules. Finally, for some modules it was possible to detect an enrichment of transcription binding sites. The binding site for *Wt1*, which is associated with neurodegeneration, was the most significantly overrepresented.

**Conclusions:**

Despite the marked differences in genetic diversity, the transcriptome structure was remarkably similar for the F_2_, HS4 and HS-CC. These data suggest that it should be possible to integrate network data from simple and complex crosses. A careful examination of the HS-CC transcriptome revealed the expected structure for striatal gene expression. Importantly, we demonstrate the integration of anatomical and network expression data.

## Background

Gene co-expression analyses have provided important insights into the functional organization of the transcriptome in several species, including yeast [1], mouse [2] and primates [3]. Co-expressed genes frequently code for interacting proteins, which in turn leads to new insights into protein function(s). Many co-expression patterns are conserved across species, suggesting the patterns are under selection pressure and therefore functional; a variety of studies have confirmed this premise [3-7].

The current study focuses on the conservation of brain gene co-expression networks from the perspective of how marked differences in genetic diversity within a species (here *Mus musculus*) affect network structure. To examine this issue, we have created a new mouse resource, a heterogeneous stock (HS) formed from the same eight inbred strains that have been used to create the collaborative cross (CC) [8]; hereafter this resource is referred to as the HS-CC. The eight inbred strains chosen as the CC founders were the following: C57BL/6J (B6), A/J (A), 129S1/SvImJ (129), NOD/LtJ (NOD), NZO/HILtJ (NZO), CAST/EiJ (CAST), PWK/PhJ (PWK) and WSB/EiJ (WSB). The choice of these strains was a balance between ensuring the greatest possible genetic diversity while at the same time including some strains (and their associated phenotypes) familiar to many biomedical scientists. Using single nucleotide polymorphisms (SNPs) as proxy for genetic diversity, these strains capture > 90% of the available genetic diversity within *Mus musculus *(see http://www.sanger.ac.uk/modelorgs/mousegenomes/). Capturing this degree of diversity is possible because of the inclusion of the three wild-derived strains: CAST (*Mus musculus castaneous*), PWK (*M.m. musculus*) and WSB (*M.m. domesticus*). To contrast with the HS-CC, we have chosen a B6×DBA/2J (D2) F_2 _intercross and the HS4, derived from intercrossing the B6, D2, BALB/cJ and LP/J strains [9]. The HS4 and HS-CC were outbred using a similar circle breeding strategy, and both are maintained as 48 families per generation. From the SNP perspective, the HS-CC is ~6 times more diverse than the F_2 _intercross and ~4 times more diverse than the HS4; the actual differences in genetic diversity will be somewhat less because of genetic drift associated with breeding the HS populations for multiple generations.

Gene expression data (Illumina WG 6.1 array) were obtained in the striatum, a relatively homogenous brain region composed largely of medium-spiny GABAergic neurons. The striatum has a key role in a wide variety of behaviours; the functions of the striatum are well understood; and a number of key striatal genes (e.g., *Drd1a *and *Drd2*) are known to have a highly variable expression [10]. The unbiased Weighted Gene Covariance Network Analysis (WGCNA) developed by Horvath and colleagues [11] was used to detect gene modules. This approach has been successfully used to analyze gene expression data related to brain cancer [12], the yeast cell cycle [1], mouse tissue [2,13], primate brain tissue [14], diabetes [15], chronic fatigue syndrome [16], plants [17] and amyotrophic lateral sclerosis [18].

The HS-CC data set was further characterized, emphasizing the fine spatial distribution of the gene modules. It has been suggested that groups of genes participating together in common biological functions may show a similar spatial pattern of expression [19]. The Allen Brain Atlas (ABA; http://www.brain-map.org/) [20] provides detailed information about the spatial distribution of thousands of genes throughout the mouse brain. For the purposes of the present study, it was possible to determine whether a particular gene is specific to the striatum and/or its subdivisions and whether its spatial distribution is uniform or clustered. In addition, the ABA interface allows quantification of the spatial similarity of two expression patterns using the NeuroBlast algorithm [21,22]. These resources were used to investigate whether groups of co-expressed genes also show spatial co-localization.

## Results

### Detectable and variable genes are preserved across populations

**Figure 1:**
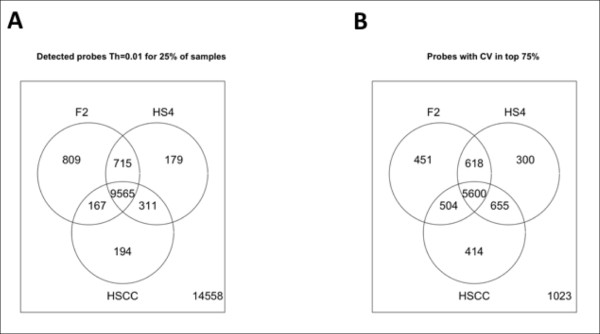
**Overlap of probe detectability and probe variability across the three data sets**. (A) Probes detected in each of the data. A set of 9565 genes were above the detection threshold in all three data sets; 14558 genes were not detected in any of the data sets. (B) Probes with high variability. A number of 5600 genes were in the top 75% in terms of coefficient of variation CV in all three data sets, and these genes were selected for network construction; 1023 were in the bottom 25% in terms of variability in all data sets.

The initial comparison of gene expression in the three mouse populations (F_2_, HS4 and HS-CC) focused on what transcripts had a detectable expression. Not all microarray probes exhibit a detectable signal because a) the target gene is not expressed, b) the expression level is below what can be reliably measured, c) the probe performs poorly, producing a false negative or d) SNP(s) within the probe sequence impair hybridization [23,24]. The SNP effect is especially important when comparing genetically diverse populations. Therefore, we removed all probes overlapping with known SNPs, as outlined in Methods. Next, the Illumina gene expression analysis package "lumi" [25] was used to assess the probability of probe expression above background. A probe was deemed to have a significant expression if the probability of being part of the background (low) distribution was less than threshold Th = 0.01 in at least a quarter of the samples. This procedure was applied to each of the three experiments after outlier removal and normalization of the data (an outline of the data pre-processing steps is available in Methods; see also Additional File 1, Figure S1 and Additional File 1, Figure S2). As shown in Figure 1A, there was a significant overlap among the three data sets for the probes meeting the threshold criteria. A total of 14558 probes did not meet the criteria for above-threshold expression in any of the data sets.

The co-expression networks are constructed on the basis of the correlated variability across individuals. Expression variability was computed by determining the coefficient of variability (CV) for the set of 9565 commonly detected probes (Figure 1A); 1023 of the probes were in the bottom quartile for all three populations. Of the remaining probes, 5600 were in the top three quartiles in all populations, illustrating the conservation of the variance structure (Figure 1B).

### Construction of gene co-expression networks

Gene co-expression networks were constructed for the three data sets following methods described previously [11]. Briefly, the power-transformed Pearson correlation coefficient between gene pairs was used to infer a measure of connection strength or topological overlap [26]. Subsequently, this measure of gene co-expression was used in an automated hierarchical clustering procedure [27], resulting in the identification of several distinct modules or groups of genes with similar expression patterns. This series of steps was used to independently detect co-expression modules in each data set, identifying 16 distinct modules in HS-CC and 13 modules each in F_2 _and HS4. Genes left unassigned to modules were denoted with the grey color. The exact number of gene modules in any network was not considered essential because the number of modules detected is highly dependent on the clustering procedure settings. The color assignment of the modules in the three different networks was arbitrary, and the same color assignment in different networks did not carry meaning, except for the unassigned genes (grey color). The genes associated with each of the modules in all three populations are listed in Additional File 2.

**Figure 2:**
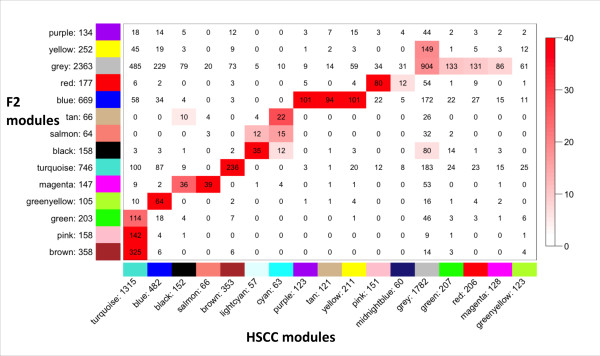
**Overlap of F_2 _and HS-CC module membership**. The numbers on the axes denote the number of genes in each module. The number in the box denotes the intersection size. The colour legend is proportional to -log(p) probability of chance overlap of same size or higher.

**Figure 3:**
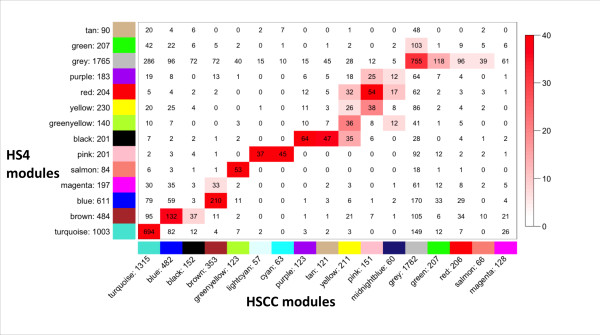
**Overlap of HS4 and HS-CC module membership**. Figure details as in Figure 2.

Comparisons of the HS-CC, F_2 _and HS4 networks are illustrated in Figures 2 and 3. The data revealed that essentially all F_2 _and HS4 modules had a counterpart in the HS-CC and vice versa (Fisher exact test), although some of the larger HS-CC modules fragmented into two or more F_2 _or HS4 modules. An examination of the unassigned "grey" genes across the three networks revealed that their identity was largely preserved.

To further quantify the level of module preservation, a matrix comparison procedure was used. Each module was described by a topological overlap matrix (TOM) with entries quantifying the level of gene pair co-expression. The matrices were compared by computing the Mantel matrix correlation [28] between the HS-CC modules and the same genes in the F_2 _and HS4 networks. A high matrix correlation signifies that the pattern of pairwise topological overlap in two different data sets was similar. Statistical significance was evaluated by repeatedly (N = 10^5^) shuffling the columns of one matrix and recomputing the correlation using the randomized matrix [29]. For the HS-CC and F_2 _comparison, the matrix correlation values ranged from 0.22 (blue module) to 0.69 (purple module). The HS-CC to HS4 comparison yielded correlation values between 0.16 (salmon module) and 0.74 (purple module). All correlation values were significant at *p *< 10^-5 ^or better, except for salmon module (*p *< 3×10^-3^). Thus, the module structure was largely preserved. This congruence suggested that each module must have functionally conserved attributes; these attributes were investigated in the HS-CC.

### Gene ontology (GO) annotation of the HS-CC modules

GO annotation [30] was used to determine if the modules had unique functional properties and/or were associated with distinct subcellular compartments (see e.g., [13]). For example, the pink module was enriched for GO biological processes that included the following: central nervous system development (Bonferroni corrected *p *< 8.6×10^-3^), regulation of neurotransmitter levels (Bonferroni corrected *p *< 8.9×10^-3^), regulation of timing of neuron differentiation (Bonferroni corrected *p *< 0.016), neuron development (Bonferroni corrected *p *< 0.036) and forebrain development (Bonferroni corrected *p *< 0.043). The red module was significantly enriched in genes corresponding to GO category behavioural fear response (Bonferroni corrected *p *< 0.0024); the tan module was enriched with genes associated with ensheathment of neurons (Bonferroni corrected *p *< 1.4×10^-4^), regulation of action potential (Bonferroni corrected *p *< 1.4×10^-3^), myelination (Bonferroni corrected *p *< 1.9×10^-2^), oligodendrocyte cell fate commitment (Bonferroni corrected *p *< 2×10^-2^), glial cell fate specification (Bonferroni corrected *p *< 2×10^-2^) and myelin assembly (Bonferroni corrected *p *< 2× 10^-2^). Only the most significant GO annotations are reported here, after taking into account the nested structure of the GO categories [31]. Bonferroni correction was applied because of comparisons against all 16 modules. A full list of significant module GO annotations is found in Additional File 3.csv.

### Proteome interactions and transcriptome co-expression

The HS-CC co-expression patterns were compared with the compiled protein-protein interactions (PPI) in the Human Protein Reference Database (HPRD) [32,33]. First, the network genes were cross-referenced with the list of HPRD gene products. Second, network genes with PPI interactions were selected, and the average topological overlap was computed. Comparing the average topological overlap of the PPI genes against an empirical distribution of random gene groups revealed that the PPI group had significantly higher topological overlap (*p *< 10^-5^). These data confirm that co-expression patterns in the transcriptome are related to interactions in the proteome, in agreement with previous results [3,34].

### Modules overlap with specific brain cell types

**Figure 4:**
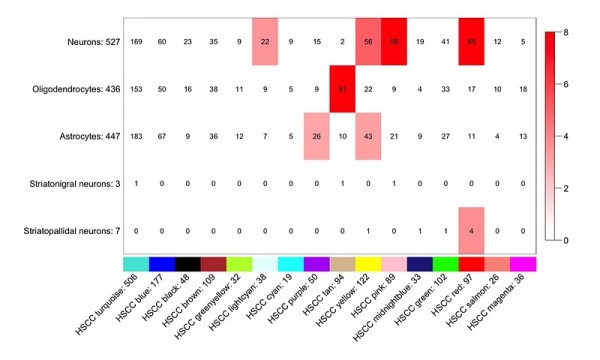
**Overlap between modules and central nervous system cell type markers**. The numbers on the y axis denote the number of genes that are markers for cell types also present in the network. The number in the box denotes the intersection size. The colour legend is proportional to -log(p) probability of chance overlap of same size or higher.

Module membership was compared against lists of genes associated with neuronal cell types [35]. Several modules (light cyan, yellow, pink and red) were enriched with neuronal cell markers (see Figure 4). The tan module was enriched with oligodendrocite specific genes, which is concordant with its GO annotation for oligodendrocyte cell fate commitment. The magenta and yellow modules contained genes associated with astrocytes. Using an additional data set that identifies genes highly specific to subcategories of striatal neurons [36], we found that the red module contains several genes associated with striatopallidal neurons.

### Spatial co-localization and transcriptome co-expression

Gene module membership was compared with the spatial distribution of the genes from the ABA [20]. Sets of co-localized genes were constructed beginning with the ten genes closest to the eigengene for each module; the eigengene modelled the representative pattern of module expression [37]. Each gene in the module was then assigned a measure of module membership "kMe", on the basis of its correlation with the eigengene. For each network module, the ten genes with highest kMe were selected. For each of these ten "seed" genes, the ABA was used to find the 250 genes with the most similar striatal spatial distribution. From this group, those present in the HS-CC network were denoted as the "co-localized" group. The Fisher exact test was used to assess the overlap between the spatially co-localized group and all members of a respective module, and Bonferroni correction was applied to correct for comparing each of the 16 modules. For eight of the modules, the overlap was signficant, with Bonferroni corrected p-values ranging from 0.01 to 6.9×10^-24^.

The extent of correspondence between co-expression and co-localization was further explored using the full set of pairwise interactions in the transcriptome with the set of pairwise spatial relationships captured in the ABA [20]. For a module, the transcriptome relationships were summarized by the topological overlap matrix. A similar size matrix for pairwise similarity in spatial profiles was constructed using the NeuroBlast algorithm [21]. Because only the top 250 most similar spatial profiles to a given gene are identified by NeuroBlast, the spatial similarity matrices were sparse. However, the Mantel test [28] still detected a moderately strong relationship between the co-expression and co-localization matrices for three of the modules: red (*r *= 0.43, *p *< 2.0×10^-5^), purple (*r *= 0.27, *p *< 3.0×10^-3^) and tan (*r *= 0.40, *p *< 10^-2^).

**Figure 5:**
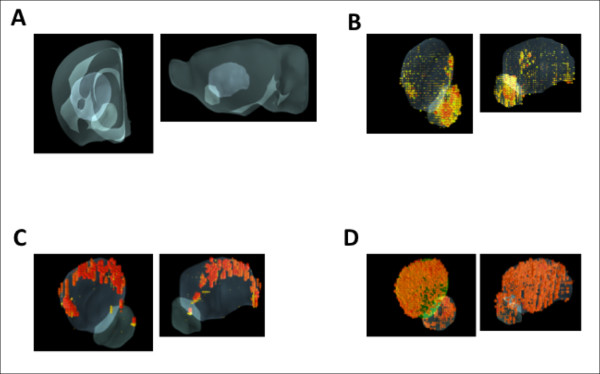
**Spatial specificity of modules**. (A) Location of the caudoputamen and nucleus accumbens within the mouse brain. Left, coronal section; right, saggital section. (B-D) The midnight blue, purple and red modules, respectively. The ten genes closest to the module eigengene are selected for each module, and their expression is superimposed. For visual clarity, only the areas with medium-high expression intensity are shown, with the darker red signifying high intensity of the hybridization signal (see Methods).

Brain Explorer [38] was used to visually inspect the spatial properties of the co-localized module genes. For the ten co-localized genes closest (in correlation) to the module eigengene, the expression levels were superimposed and plotted over the extent of the striatum (see Figure 5). A few of the co-localized modules displayed distinctly localized patterns of spatial expression. The high expression of the midnight blue module (Figure 5B) appeared largely restricted to the nucleus accumbens, while the purple module (Figure 5C) displayed a very distinctive dorsal tier pattern of expression within the caudate putamen. A more typical pattern is that of the red module (Figure 5D) which appeared highly expressed through most but not all of the area of the striatum. The GO categories enriched in the midnight blue, purple and red modules are found in Additional File 3.

### Transcriptional regulatory analysis of gene modules

Module-specific genes were examined for overrepresentation of transcription factor binding sites (TFBS). The strategy employed is detailed in Methods. Using a false discovery rate threshold of 0.1, we identified 9 TFs preferentially affecting 5 gene modules (Table 1). The most highly enriched TF was Wilm's tumour suppressor (Wt1) in the pink module (*p *< 3×10^-5^); this module is modestly enriched in GO categories associated with the regulation of transcription and neuronal development (see Additional File 3) and has also been associated in the literature with distinct neuropathological conditions such as Alzheimer's disease [39]. The two modules (midnight blue and purple) that show distinct patterns of expression (see Figure 5) were not associated with TF enrichment. The blue and red modules were enriched in Specificity protein (Sp)-1, which is known to have a role in the regulation of striatal gene expression (e.g., [40]).

**Table 1:** List of transcription factors (TFs) affecting specific module genes at a threshold of false discovery rate (FDR) = 0.1 of enrichment compared with the rest of the network.

Module	TF	Raw p-value/FDR	TFBS/genes in module
Pink	Wt1	3e-05/0.00543	89/151
Black	LRH1	0.001/0.0972	32/152
Black	Pax-6	0.00057/0.0972	73/152
Blue	ZF5	0.00001/0.0019	248/482
Blue	Sp1	0.00097/0.0614	182/482
Blue	HIC1	0.00064/0.0608	60/482
Red	Sp1	0.0004/0.0366	87/206
Red	CP2	0.00016/0.02928	97/206
Turquoise	CETS1p54	2e-05/0.0039	419/1315

## Discussion

Previous studies have reported preservation of gene co-expression modules across mouse strains [41] and even across species [14]. The level of preservation or divergence is usually quantified by the amount of overlap of gene modules from different networks. While this approach has been proven useful and we also employed it in the present study (see figures 2 and 3), it is highly dependent on the clustering procedure. We therefore employed an additional, complementary approach of quantifying the level of preservation: the Mantel test of correlation between two matrices [28]. This method has been used extensively in quantifying relationships between genetic, geographic and environmental distances [42], and more recently has been adapted to quantification of differentially expressed genes [29]. If topological overlap between genes is preserved, this is detected as high correlation between the respective TOM matrices. Additionally, the Mantel test can be used for comparing any similar rank matrices, as we illustrate by quantifying the relationship between topological overlap and spatial co-localization, both of which can be represented as matrices of pairwise interactions. Overall, the data illustrate that, despite a > 6-fold difference in genetic diversity (HS-CC versus F_2_), the overall module structure of the striatal networks was preserved. Intuitively, this congruence may seem obvious because the striatum performs a similar function in all three populations. However, there was a concern that the marked increase of genetic diversity in the HS-CC, as compared with the F_2 _and HS4, would have such marked effects on gene expression variance that the overlap of the networks and their modules would be difficult to detect. While the topological overlap matrices did suggest that there are some differences and that some modules agree significantly better than others, the overall structure remained intact.

The HS-CC data illustrate that relatively sophisticated module details can be detected with relatively modest sample sizes. Confirming the results of others [3], it was possible to detect modules that were enriched in genes associated with neurons (light cyan, yellow, pink and red), oligodendrocytes (tan) or astrocytes (magenta and yellow). Only the yellow module showed an overlap. These data suggest that there are sufficient numbers of each cell type (and thus statistical power) to allow the clustering procedures to detect cell-type specific modules. A similar argument could be used to explain our ability (albeit somewhat limited) to detect unique spatial localizations for some modules. A majority of genes in our network were categorized in ABA as having uniform spatial distribution. However, the striatum is a complex structure with distinct subdivisions, some of which have been associated with specific behavioural states [43]. Even the most ubiquitous cell type within the striatum, the medium-spiny neuron, displays distinct morphological characteristics based on its spatial position, for instance in the core or shell of the nucleus accumbens [43]. Microarray samples are an amalgam of cell types from many distinct anatomical substructures. While gene expression studies have been instrumental in mapping behaviour into specific physiological processes, progress is hampered by the lack of more specific information about the cell types and anatomical substructures directly involved in patterns of gene co-expression. Our study leverages the vast amount of information available in mouse atlases such as ABA. This approach can be further enhanced by information emerging from studies using laser-captured neurons [44].

The detection of transcription factors (TFs) specific to distinct module genes provides a candidate mechanism for generating the co-expressed patterns of gene expression. We were unable to align the distinct patterns of expression in the midnight blue and purple modules with specific TFs. The purple module is of particular interest given the overexpression of the module genes in the nucleus accumbens and the role the accumbens has in a variety of behaviours including reward, reinforcement and drug abuse [43]. Understanding the factors associated with co-expression in this region has the potential to lead to new molecular-based treatments. The red module, which is associated with gene expression throughout the striatum (Figure 5D), is enriched in the Specificity protein 1 (Sp1) TF; the blue module also showed enrichment in Sp1. Sp1 is known to affect pathways associated with neuronal survival and death [45], and the dysregulation of SP1 has been associated with Alzheimer's disease [46]. The antibiotic mithramycin binds to G-C rich DNA sequences to inhibit the binding of SP1 [47]. Previous work [40] has shown that the administration of mithramycin blocks the striatal toxicity associated with chronic methamphetamine administration. It is of interest to speculate that this effect may be mediated through an influence on the genes within the red and blue modules.

## Conclusions

We here present an integrative approach to the analysis of mouse brain transcriptome data. The modular structure of the striatum transcriptome is largely preserved despite large genetic differences among the HS-CC, F_2 _and HS4 populations. Gene co-expression modules have spatial co-localization in some cases. A small set of TFs has a strong overabundance specific to distinct modules. These TFs have been previously associated with changes in behaviour or neuropathology, indicating that using a network-based comparison holds strong promise for the elucidation of underlying regulatory mechanisms. Finally, to our knowledge this is the first report on the application of gene network analyses to HS populations. HS populations have proven useful for the fine mapping of quatitative trait loci (QTL) and for the integration of QTL and gene expression data [9,48-53].

## Methods

### Animals

#### Breeding the HS-CC Mice

Males and females of the 8 parental strains (B6, CAST, NOD, 129, NZO, PWK, A and WSB) were obtained from The Jackson Laboratory. The strains were randomly assigned a letter from A to H; the order of assignment was the order noted above. The goal of the breeding strategy described below was to create a small panel (32 families) of the HS-CC mice; for such a small panel, a completely balanced breeding design is not possible. At G_1_, the following reciprocal F_1 _hybrids were formed: A×B, B×A; C×D, D×C; E×F, F×E; G×H, H×G. At G_2_, the following reciprocal 4-way crosses were formed: AB×CD, CD×AB; BA×DC, DC×BA; EF×GH, GH×EF; FE×HG, HG×FE. At G_3_, 32 unique 8-way cross families were formed: ABCD × EFGH, ×GHEF, ×FEHG, ×HGFE...........HGFE × ABCD, ×CDAB, ×BADC, ×DCBA. Each family was bred in duplicate. Of the 64 matings, 61 had litters; the three matings without litters were CDAB × EFGH, EFGH × CDAB and CDAB × HGFE. Thus, all 32 of the planned families were formed. Beginning with G_4_, the families were outbred using a circle breeding design--a male from family 1 was bred to a female from family 2 and so on. At G_6_, the colony was expanded to 48 families by breeding a male from family 1 to a female from family 17 and so on. At G_12_, one male and one female from each family were chosen for striatal gene expression analysis.

#### Breeding the HS4 Mice

Males and females of the 4 parental strains (B6, D2, C and LP) were obtained from The Jackson Laboratory. At G_1_, the 12 possible reciprocal F_1 _hybrids were formed, followed at G_2 _by forming the 48 possible reciprocal 4-way crosses. The 4-way crosses were then outbred following a similar design to that noted for the HS-CC. At G_19_, one male and one female from each family were randomly chosen for striatal gene expression analysis. Details of the sample preparation are found in [9]. Based on RNA quality, on maximizing family diversity and on gender neutrality, 64 samples were chosen for gene expression analysis. High-quality data were obtained for 54 samples.

#### Breeding the F_2 _Mice

Male and female B6 and D2 mice were obtained from The Jackson Laboratory. The reciprocal F_1 _hybrids were formed, followed by the formation of the 4 possible reciprocal F_2 _hybrids. Eight males and females were randomly selected from each of the reciprocal crosses. From the 64 samples, high-quality expression data were obtained for 56 samples.

All animal care, breeding, and testing procedures were approved by the Laboratory Animal Users Committees at the Veterans Affairs Medical Center, Portland, OR 97239, and the Oregon Health & Science University, Portland, OR 97239.

### Gene expression data processing

Gene expression data were obtained from the striatum using the Illumina WG 6.1 array exactly as described by the manufacturer. Data were imported into the R application environment (http://www.r-project.org) using the lumi package [25]. Samples that were more than two standard deviations away from the mean inter-array correlation (IAC) [3] were not used in this study. This procedure was repeated three times resulting in stabilization of IAC and reduction of the data sets from 94 to 87 samples (HSCC), 60 to 56 samples in F_2 _and 54 to 47 samples in the HS4.

Strip-level quantile normalization [54] was performed using a modified version of the procedure available in the lumi package (see Additional File 1, Figure S2). Next, data were culled for any probes that did not have an entrezID and also any probe that overlapped with known SNPs in any of the founding populations, using the publicly available Wellcome Trust Sanger Institute database of known polymorphisms (http://www.sanger.ac.uk/resources/mouse/genomes/). Further removed from analysis was any probe unlikely to be reliably detected [23,24], using the detectionCall procedure available in the lumi R package. Using a cutoff threshold of 0.01, all probes not expressed in at least a quarter of the samples were removed. Finally, to reduce the size of the data to a level suitable for subsequent network analysis and to reliably compute the correlation between probe levels, we eliminated probes with variability in the bottom 25% of any data set, as measured by the lumi function estimateLumiCV. This resulted in a set of 5600 probes common across the three data sets, which were subsequently used in the construction of the gene co-expression networks (see Figure 1B).

### Construction of the gene co-expression networks

For each of the three data sets, we performed a series of steps for constructing a gene co-expression network, as outlined in [55], using the WGCNA software package available as an R package [11]. First, the absolute value of the Pearson correlation coefficient was computed for all pairs of genes in a data set. The Pearson correlation matrix was subsequently transformed into an adjacency matrix A using a power function. The connection strength a_ij _between probes x_i _and x_j _then becomes a_ij _= |corr(x_i_, x_j_)|^β^; β = 6 was used based on the scale-free topology criterion [55].

Modules are groups of genes with high 'topological overlap' [55,56]. The topological overlap between two genes i, j was computed as $\omega_{ij}=\frac{l_{ij}+a_{ij}}{\min\left\{k_{i},k_{j}\right\}+1-a_{ij}}$, where $l_{ij}=\sum_{u}a_{iu}a_{uj}$ represents the number of genes connected to both gene i and gene j, while *u *indexes all the genes in the network. Using the topological overlap measure as opposed to the raw adjacency values minimizes the effects of spurious connection strengths between any two genes. The network modules were defined as branches of the clustering tree resulting from the dissimilarity matrix *d*_*ij *_= 1-*ω*_*ij*_. We used the "dynamic tree cut algorithm" [27], which takes advantage of the internal structure of the dendrogram in cutting the branches and identifying modules.

### GO annotation of gene modules

Each modules gene was tested for GO enrichment [30] using the GOstats R package [57]. Because of the nested structure of the GO terms, we employed the graph decorrelation procedure suggested by [31]. The resulting p-values were further adjusted using the Bonferroni procedure, which accounts for comparison against multiple modules [13].

### Proteome interactions and transcriptome co-expression

The gene network co-expression patterns were compared with a manually compiled protein-protein interactions (PPI) database retrieved from the Human Protein Reference Database (HPRD) [32,33]. Using EntrezIDs, we selected the network genes also present in the list of HPRD gene products. The network genes with PPI interactions were selected and the average topological overlap was computed, as was the average topological overlap for gene groups of same size but randomly selected (N = 10^5^). Statistical significance was assessed by counting the number of times random gene groups displayed higher topological overlap (in this case none).

### Quantification of spatial co-localization

The ABA quantifies the local intensity of gene expression in an image by using individual cubes of 200 μm^3 ^and computing for each the expression energy:

$E(C)=\frac{\sum_{p\in C}M(p)\times I(p)}{\left|C\right|}$, where C is the set of pixels that intersect a cube, M(p) is a binary mask with the value 1 for pixels intersecting a cube, and I(p) is the greyscale value of the ISH image. The spatial correlation between two image series X, Y is then computed as the Pearson correlation coefficient:

$CC(X,Y)=\frac{N\sum XY-\sum X\sum Y}{\sqrt{\left[N\sum X'-\left(\sum X\right)\right]\left[N\sum Y'-\left(\sum Y\right)\right]}}$, where the summation is over all N cubes in the domain.

The web interface of the ABA allows the retrieval of the 250 genes with highest spatial correlation to a gene of interest. We restricted the spatial extent of computing the spatial correlation to the striatum.

To find the most representative members of each module, the module eigengene, which is the first principal component of the matrix representing all the expression patterns of module genes [37] was computed. The correlation between the expression pattern of each gene and the module eigengene results in a measure of the strength of module membership. For each module, the top 10 genes ranked in terms of eigengene-based module membership were selected; subsequently, ABA interface was used to retrieve the 250 genes most spatially correlated to these top 10 genes.

To perform the Mantel test for correlation between co-expression and co-localization, a square matrix was constructed with entries quantifying the strength of spatial correlation between the genes, with NA denoting unavailable information due to the ABA restricting the results to only the top 250 most similar genes. This square matrix was used in the Mantel test for correlation between co-localization and co-expression, using the R package "ncf" (http://cran.r-project.org/web/packages/ncf).

### Detection of overrepresented TFBSs within the gene modules

For the detection of TFBSs within modules, the Promoter Analysis and Interaction Network Tool (PAINT) was used [58]; PAINT is a software tool available online (http://www.dbi.tju.edu/dbi/tools/paint/index.php), which connects with the TRANSFAC database [59]. Using the MATCH algorithm [60] and position weight matrix descriptions of binding sequences, the upstream region of each gene is searched for TFBSs. Our search focused on the 2000 base pairs upstream from putative start sites, used the "minimize false positives" setting and selected only the TFBSs that had a perfect match to the 5 base pair core sequence in the transcriptional regulatory element. Once the putative TFBSs were identified, PAINT was used to compare each module for overabundance of specific TFBS against the rest of the network, with statistical significance assessed using the Fisher exact test. The raw p-values were further adjusted due to multiple comparisons [61] using a false discovery rate approach [62].

## Authors' contributions

BM, DO and PD were responsible for sample preparation, collecting the gene expression data and the initial gene expression analyses. OI, PD, NW, JB and SM were responsible for most of the detailed analyses. OI was solely responsible for the alignment of the spatial and co-expression data and prepared all drafts of the manuscript. PD assisted in the image data retrieval from the Allen Brain Atlas web site. SM and RH edited the manuscript and provided overall oversight of the project. Dr. Kristin Demarest independently reviewed the manuscript prior to publication, and her contributions are gratefully acknowledged. Dr. Armand Bankhead provided help in the HPRD data retrieval. All authors have read and approved the final manuscript.

## Supplementary Material

Additional file 1 **This file contains additional figures detailing the data processing steps, including outlier sample removal and strip level normalization**. Click here for file

Additional file 2 **This file contains all 5600 genes selected for the network analysis**. The genes are identified by EntrezID, gene symbol as well as nuId, an unique identifier for Illumina probes [63]. The module color assignment for each of the three networks is provided for each gene. Click here for file

Additional file 3 **This file contains all the GO annotations for the 16 module genes detected in the HS-CC network**. Click here for file
