# Supplementary material for: Genetic diversity and striatal gene networks: focus on the heterogeneous stock-collaborative cross (HS-CC) mouse
Source: BMC Genomics. 2010 Oct 19;11:585. doi: 10.1186/1471-2164-11-585 (PMC3091732; doi:10.1186/1471-2164-11-585)
Supplement: Additional file 1 — This file contains additional figures detailing the data processing steps, including outlier sample removal and strip level normalization. [file 1471-2164-11-585-S1.DOC]

# Genetic diversity and striatal gene networks: focus on the heterogeneous stock–collaborative cross (HS-CC) mouse

Ovidiu D. Iancu^1,§^, Priscila Darakjian^1^, Nicole A.R. Walter^1^, Barry Malmanger^1^, Denesa Oberbeck^1^, John Belknap^1.2^, Shannon McWeeney^3,4^ and Robert Hitzemann^1,2^

^1^Department of Behavioral Neuroscience, Oregon Health & Science University, Portland, OR 97239, USA

^2^Research Service, Veterans Affairs Medical Center, Portland, OR 97239, USA

^3^Division of Biostatistics, Public Health & Preventative Medicine, Oregon Health & Science University, Portland, OR 97239, USA

^4^Division of Bioinformatics and Computational Biology, Medical Informatics & Clinical Epidemiology, Oregon Health & Science University, Portland, OR 97239, USA

^§^Corresponding author: ODI

Email addresses: iancuo@ohsu.edu, darakjia@ohsu.edu, waltern@ohsu.edu, malmange@ohsu.edu, oberbeck@ohsu.edu, belknajo@ohsu.edu, mcweeney@ohsu.edu, hitzeman@ohsu.edu

**Supplementary figures for gene expression data processing**

In order to remove samples with questionable expression data, we computed the inter array correlation (IAC) [1]. The IAC is simply the correlation between samples across all probes. Next, the average of this correlation is computed for each sample and these quantities are normalized. The samples that lie more than two standard deviations from the mean are considered outliers and are removed from the data. This procedure was repeated three times. This step is illustrated bellow for the HS-CC; essentially similar steps were taken in HS4 and F2.


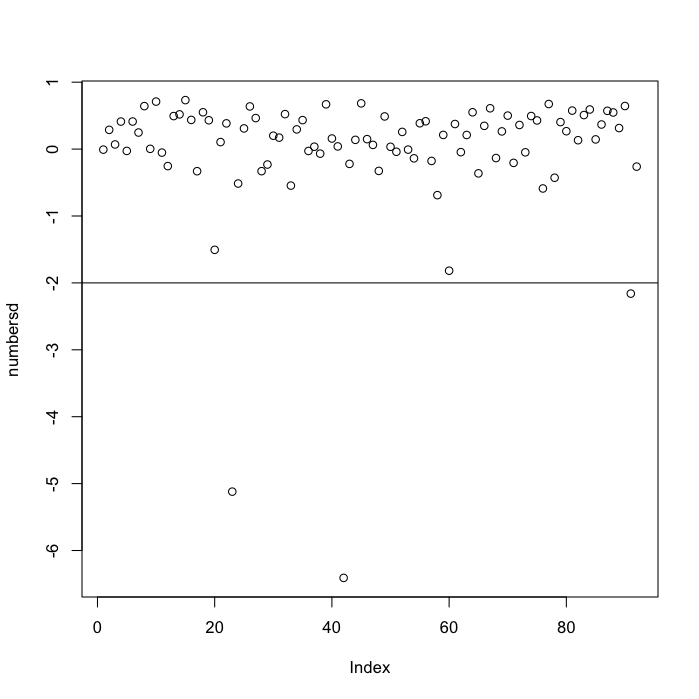


Figure S1 The normalized IAC for the HS-CC data. Sample indexes are on the x axis and their normalized IAC is on the y axis. Samples bellow the two standard deviation line are removed from the data.

Following a recent study of the effect of two different strips on each Illumina v1 array [2], we performed strip level quantile normalization using a modified version of the procedure available in the lumi package [3].


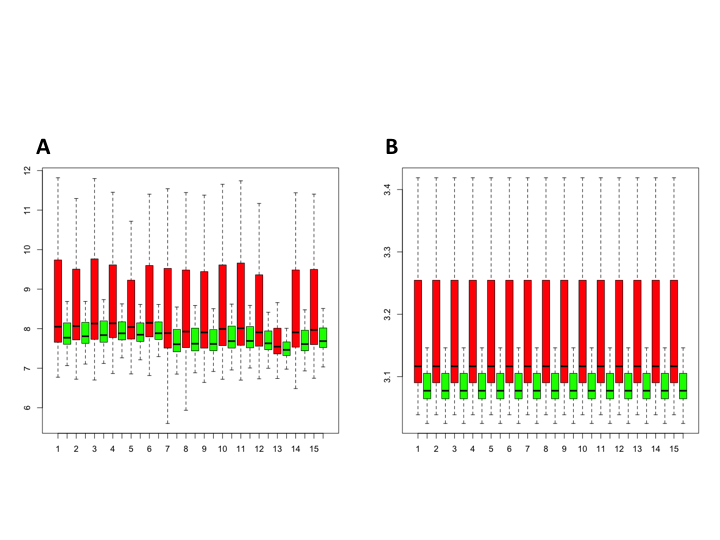


Figure S2 Strip normalization of the HS-CC expression data. A: Boxplots of 15 samples . Red boxes contain the first strip probes, green boxplot the second strip. We can observe large disparities in the mean and the spread of the expression levels between the two strips. Sample 13 is an outlier as detected IAC based procedure. B: Data in A after strip-based quantile normalization.

1. Oldham MC, Konopka G, Iwamoto K, Langfelder P, Kato T, Horvath S, Geschwind DH: **Functional organization of the transcriptome in human brain**. *Nat Neurosci* 2008, **11**(11):1271-1282.

2. Shi W, Banerjee A, Ritchie ME, Gerondakis S, Smyth GK: **Illumina WG-6 BeadChip strips should be normalized separately**. *BMC Bioinformatics* 2009, **10**:372.

3. Du P, Kibbe WA, Lin SM: **lumi: a pipeline for processing Illumina microarray**. *Bioinformatics* 2008, **24**(13):1547-1548.
